# Global research trends between gut microbiota and lung cancer from 2011 to 2022: A bibliometric and visualization analysis

**DOI:** 10.3389/fonc.2023.1137576

**Published:** 2023-02-23

**Authors:** Haitao Chen, Yuebiao Lai, Chenxiao Ye, Changhong Wu, Jiali Zhang, Zewei Zhang, Qinghua Yao

**Affiliations:** ^1^ Department of Integrated Chinese and Western Medicine, The Cancer Hospital of the University of Chinese Academy of Sciences (Zhejiang Cancer Hospital), Institute of Basic Medicine and Cancer (IBMC), Chinese Academy of Sciences, Hangzhou, Zhejiang, China; ^2^ Integrated Traditional Chinese and Western Medicine Oncology Laboratory, Key Laboratory of Traditional Chinese Medicine of Zhejiang Province, Hangzhou, Zhejiang, China; ^3^ Community Health Service Center of Louta Town, Hangzhou, Zhejiang, China; ^4^ The First Clinical College of Zhejiang Chinese Medical University, Hangzhou, Zhejiang, China; ^5^ The Second Clinical College of Zhejiang Chinese Medical University, Hangzhou, Zhejiang, China; ^6^ Department of Hepatobiliary and Pancreatic Surgery, The Cancer Hospital of the University of Chinese Academy of Sciences (Zhejiang Cancer Hospital), Hangzhou, Zhejiang, China; ^7^ Key Laboratory of Head & Neck Cancer Translational Research of Zhejiang Province, Hangzhou, Zhejiang, China

**Keywords:** gut microbiota, lung cancer, bibliometric, CiteSpace, VOSviewer, visualization

## Abstract

**Background:**

An increasing number of studies have found that the gut microbiota was related to the occurrence and development of lung cancer. Nonetheless, publication trends and research hotspots in this field remain unknown. The study aimed to perform a bibliometric analysis to systematically identify publication trends and research hotspots in the field of gut microbiota and lung cancer research within a 12-year panorama.

**Methods:**

Publications related to the gut microbiota and lung cancer between 1 January 2011 and 25 October 2022 were retrieved from the Web of Science Core Collection (WoSCC) database. The online analytic tool of the WoSCC was used to analyze various bibliometric parameters. The bibliometrics website, CiteSpace, and VOSviewer were used to identify research trends and hotspots.

**Results:**

A total of 375 publications related to the gut microbiota and lung cancer were extracted from WoSCC and identified for analysis. The number of annual publications has grown rapidly since 2018 and reached a peak in 2022. China was the most prolific country in this field, with 120 publications, followed by the United States (114), with the highest H-index of 31. Additionally, France ranked the highest with an average of 133 citations, while the leading institution and journal were the Unicancer and the *International Journal of Molecular Sciences*, respectively. Interestingly, Routy Bertrand was the most prolific author and also the most cited author in terms of H-index and citations. Reference and keyword burst detection indicated that the research hotspots mainly included 1) the gut microbiota directly affects the efficacy of immunotherapy for lung cancer, 2) the application of different gut bacteria on lung cancer, and 3) the mechanism of the gut microbiota on lung cancer.

**Conclusion:**

The findings of this study revealed the general publication trends and evolving research hotspots in the field of gut microbiota and lung cancer at a global level. The research hotspots focused on the clinical application of the gut microbiota combined with immunotherapy in lung cancer and its mechanism. The findings of this study provide new perspectives on the field, which may shed light on a beneficial impact on further etiological studies, diagnosis, and treatment for lung cancer.

## Introduction

Globally, lung cancer is currently the leading malignancy in terms of incidence and mortality among cancers, while the annual incidence rate increased continuously (1). Epidemiological surveys demonstrated that there were about 2.1 million new cases of lung cancer in 2018, with an estimated 1.8 million deaths, accounting for nearly one-fifth (18.4%) of cancer deaths (2, 3). Hence, lung cancer has seriously affected people’s quality of life and posed a certain socioeconomic burden; hence, the prevention and treatment of lung cancer are paramount.

An increasing number of studies have found that the development of many diseases was related to dysbiosis of the gut microbiota, including but not limited to inflammatory bowel disease (4), atrial fibrillation (5), and Parkinson’s disease (6). Notably, numerous previous studies identified that the change in the gut microbiota was associated with lung cancer progression (7–9). Dysbiosis of the gut microbiota may promote the occurrence and development of lung cancer by regulating metabolic pathways, suppressing immune cell function, producing pro-inflammatory factors, and promoting immune escape (10–12). Therefore, the gut microbiota plays a crucial role in the progression of lung cancer. Meanwhile, these studies also suggested that the gut microbiota is a potential marker and therapeutic target for lung cancer.

As a well-established method for analyzing publication information, bibliometric analysis has been widely used in different research areas (13–16), specifically the identification of bibliometric relevant parameters, such as core scholars/institutions/countries and their collaborative associations, keyword co-occurrence, and bursts analysis, which can significantly contribute to revealing the current status, hotspots, and research trends over time in given research fields (17). To the best of our knowledge, although the number of annual publications continues to grow rapidly, no in-depth bibliometric analysis has been conducted in this field. Therefore, this study aims to systematically reveal the research trends and hotspots in the field of gut microbiota and lung cancer over the past 12 years by using bibliometric analysis for the first time, providing a new perspective for future research in this field.

## Methods

### Data source and search strategy

Web of Science Core Collection (WoSCC) is widely used for visualization and quantitative analyses, which is the most authoritative citation-based database with the function of a powerful index (18–20). All data were retrieved from the WoSCC in this study, with the timespan from 1 January 2011 to 25 October 2022.

The search strategy employed was as follows: TS=(((intestin) OR (gastrointestin) OR (gut) OR (gastro-intestin)) AND ((Microbiot) OR (Microbiome) OR (Flora) OR (Microflora) OR (Bacteria) OR (antibiotic) OR (probiotic) OR (prebiotic) OR (dysbiosis))) AND (lung) AND ((cancer) OR (tumor) OR (tumour) OR (carcinoma) OR (neoplasm)). Two authors (HT Chen and YB Lai) completed the data extraction on 26 October 2022 to reduce the deviation caused by data extraction. Any disagreement was resolved by discussion or by seeking the assistance of a third author (QH Yao). Additionally, only articles or review articles were included, while there were no strict language restrictions.

### Data collection and bibliometric analysis

Different file formats were downloaded from the Web of Science website and exported for analysis. Analysis metrics mainly included the annual number of publications, number of total citations, average citations per publication (CPP), country, institution, journal, keywords, authors, Hirsch index (H-index) (21), the impact factor (IF; 2021), and the category quartile. Additionally, the bibliometrics website (http://bibliometric.com/) was used to generate a visual cooperation map of the countries/regions, herein identifying the countries that cooperate most closely with each other (22, 23).

CiteSpace (Version 5.6.R3) was regarded as an excellent visualization tool invented by Professor Chaomei Chen, which was widely used for countries, journals, dual-map analysis, institutions, cited references, and timeline view and for detecting the keywords with strong citation bursts (24, 25). The parameters of CiteSpace were set as follows: time slicing was from 2011 to 2022, years per slice (1); selected one node type at a time; and selection criteria were the top 50 objects. VOSviewer (Version 1.6.18) was used to generate the visual map of co-authorship of authors, citation of authors, citation of references, and co-occurrence of keywords (26, 27). Additionally, the keywords that appear at least 10 times were used to analyze the hotspots in this field.

## Result

**Figure 1:**
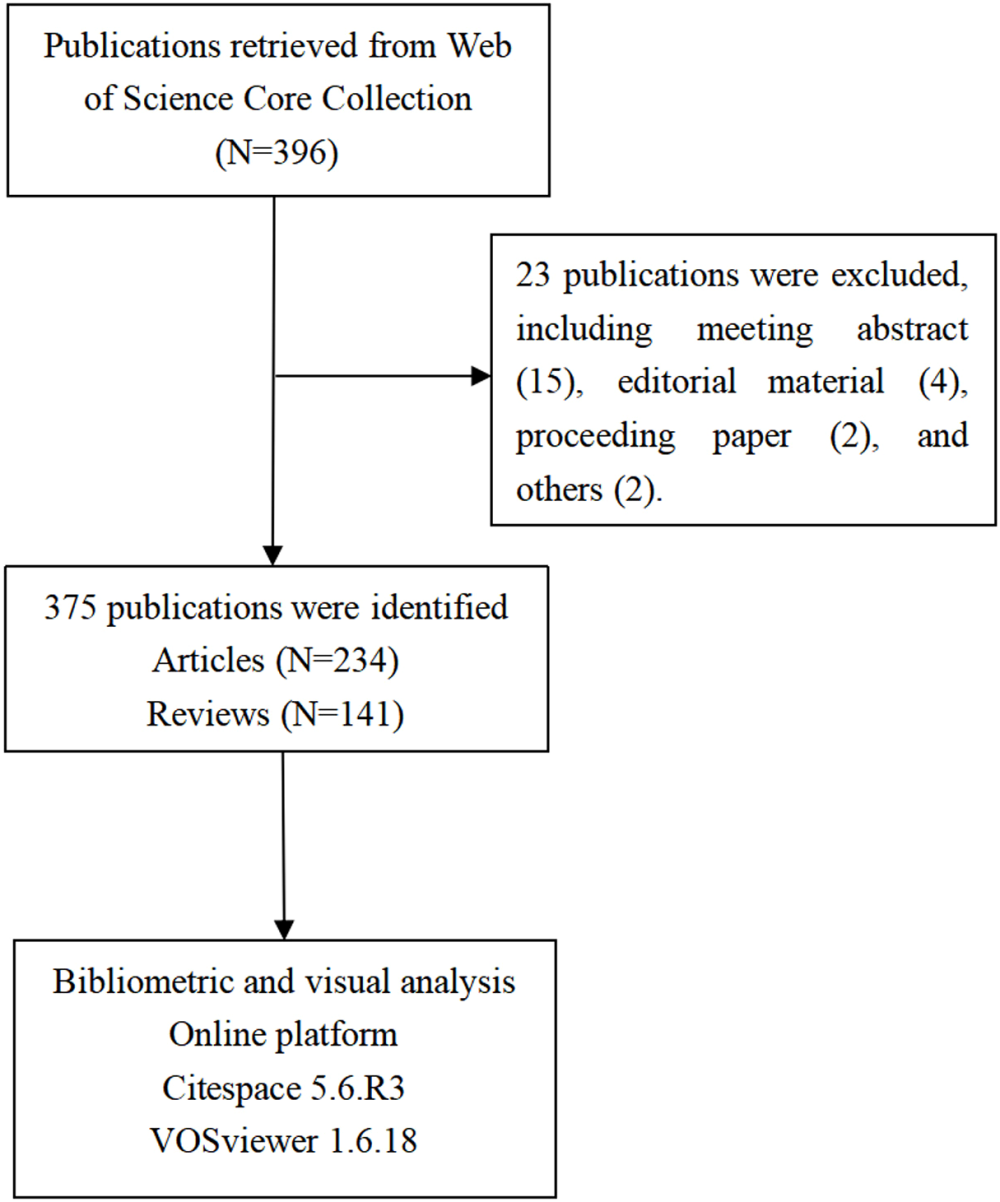
Flowchart of the study strategy.

A total of 396 publications related to the gut microbiota and lung cancer were extracted from WoSCC, with the timespan from 2011 to 2022. Excluding publications that were non-articles or reviews, such as meeting abstracts (N = 23), 375 publications were ultimately included for scientometric and visual analyses. The specific publication screening flowchart is shown in **Figure 1**.

### Global trends of publication outputs and citations

**Figure 2:**
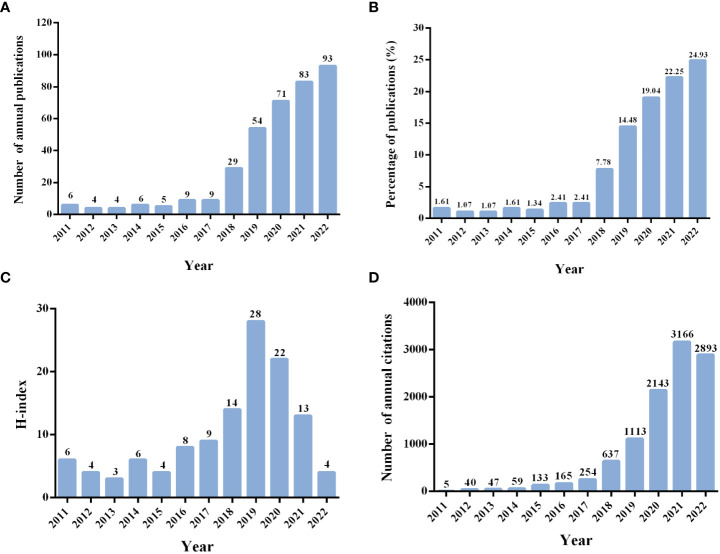
**(A)** Number of annual publications. **(B)** Annual percentage of the published publications. **(C)** Annual H-index of the publications. **(D)** Number of annual citations.

The characteristics of 375 publications are demonstrated in **Figure 2**. As shown in **Figures 2A, B**, the number of annual publications was small in the first 7 years, with no more than 10 publication outputs per year, accounting for 11.47% of 375. However, the number of annual publications has grown rapidly since 2018 (N = 29, accounting for 7.78% of 375) and reached a peak in 2022 (N = 93, accounting for 24.93% of 375), which indicated that the researchers had invested more interest and outputs in this field in recent years. There were some fluctuations in the value of the H-index over the past 12 years, and the highest year was 2019 (**Figure 2C**). Additionally, to the search date, all publications were cited 10,770 times, with a CPP of 28.72 and an H-index of 50. The top 5 cited years were identified, including 2021, with the number of annual citations 3,166 times, followed by 2022 (2,893 times), 2020 (2,143 times), 2019 (1,113 times), and 2018 (637 times) (**Figure 2D**).

### Contributions of top 10 productive countries/regions

**Figure 3:**
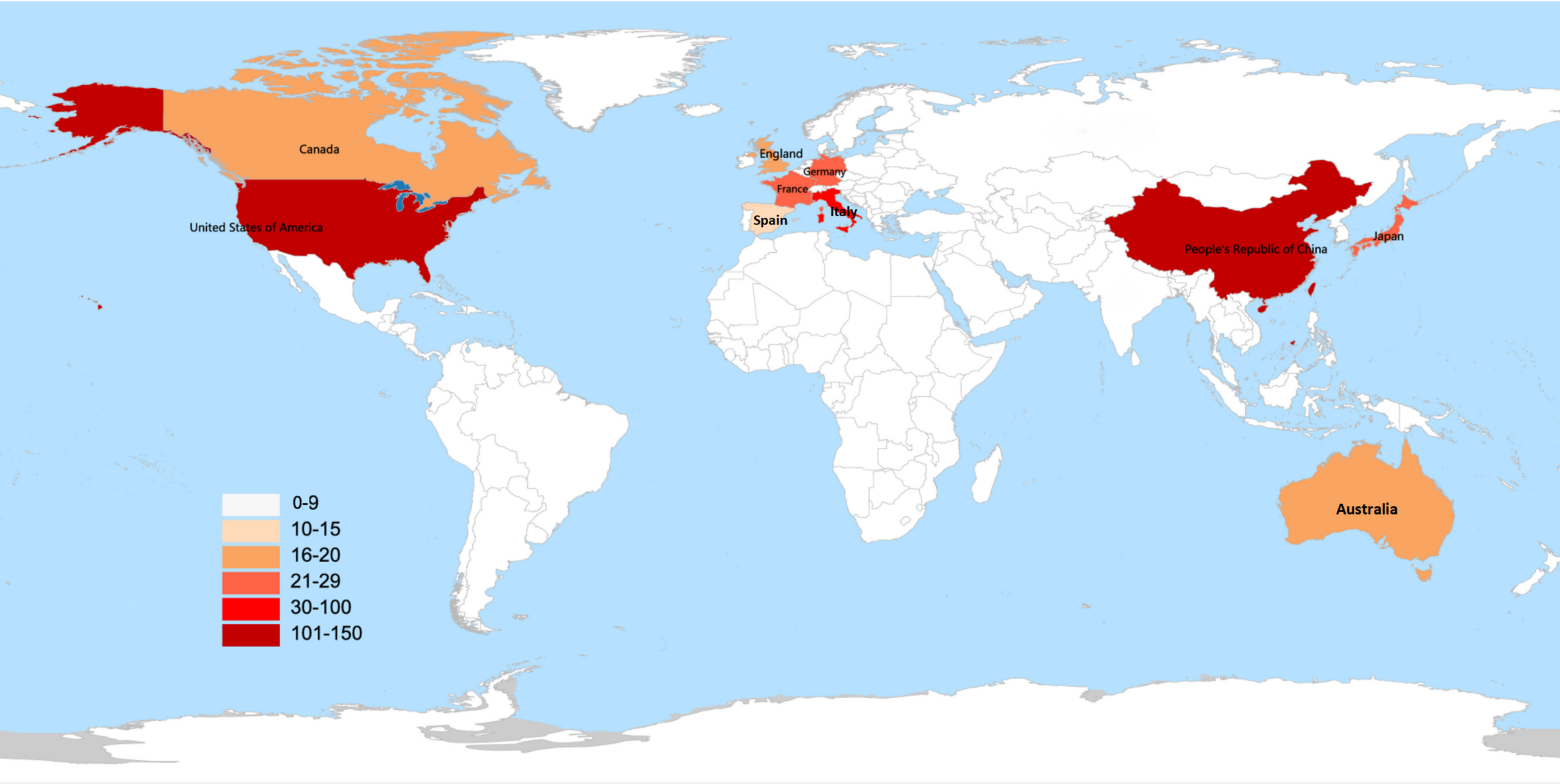
World map based on the total publications of the top 10 countries/regions. The different colors in the map represent the total number of publications, with the darker red color representing the higher number of publications.

**Figure 4:**
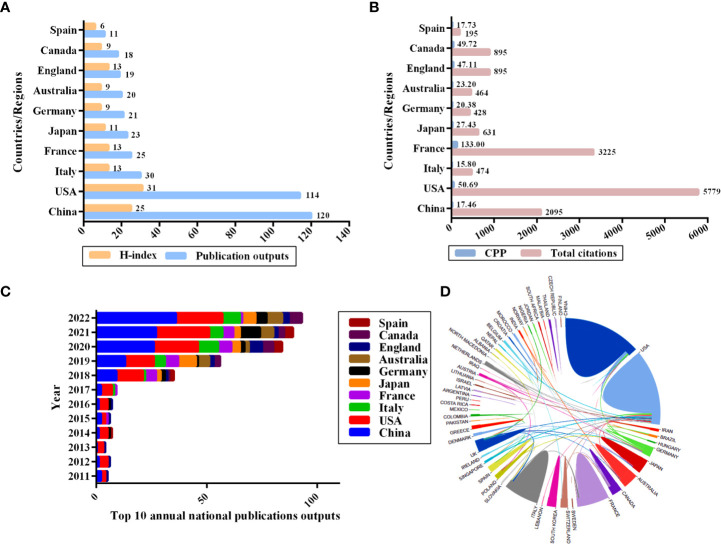
Top 10 productive countries related to the gut microbiota and lung cancer from 2011 to 2022. **(A)** The number of publications and H-index. **(B)** The total number of citations and average citations per publication. **(C)** The average annual number of national publications. **(D)** An international collaboration between countries. The countries were labeled using different colors, and the links represent international collaborations.

All 375 publications were distributed covering 60 countries/regions. The world map based on the top 10 countries in terms of the number of publications issued related to the gut microbiota and lung cancer is shown in **Figure 3**. The different colors on the map represent the total number of publications, with the darker red color representing a higher number of publications. China was the most prolific country in this field, with 120 publications, followed by the United States (114), Italy (30), France (25), and Japan (23), while the United States had the highest H-index (31) (**Figure 4A**). When the country citations were analyzed, the United States, with 5,779 citations, had the highest total number of citations, followed by France (3,225), China (2,095), Canada (895), and the United Kingdom (895) (**Figure 4B**). As shown in **Figure 4B**, among the top 5 countries in CPP, France ranked the highest with an average of 133 citations, followed by the United States (50.69), Canada (49.72), the United Kingdom (47.11), and Japan (27.43). In addition, the number of annual publications had increased significantly in China, the United States, and France since 2018. China surpassed the United States as the country with the most publications in this field post-2020 (**Figure 4C**). Furthermore, the visual map of international collaboration between the different countries was also captured. The United States maintains the strongest partnerships with other countries in this field, working most closely with China, followed by Germany, Australia, France, and Singapore (**Figure 4D**). However, cooperative relationships among other countries were fragile.

### Analysis of the leading institutions and journals

A total of 941 institutions were active in this field, and the top 10 institutions in terms of publications are listed in **Table 1**; most of them were from France, followed by the United States and China. The most prolific institution was Unicancer, with 16 papers, followed by Udice French Research Universities (with 15 papers), Harvard University (with 15 papers), and Institut National De La Santé Et De La Recherche Médicale Inserm (with 14 papers). Harvard University had the highest H-index (11), while Gustave Roussy occupied the highest contribution of CPP (272.00).

**Table 1:** The top 10 institutions in the field of gut microbiota and lung cancer from 2011 to 2022.

Rank	Institutions	Counts	% of 373	Citations	CPP	H-index	Location
1	Unicancer	16	4.29	3,145	196.56	10	France
2	Udice French Research Universities	15	4.02	3,135	209	10	France
3	Harvard University	15	4.02	459	30.6	11	USA
4	Institut National De La Santé Et De La Recherche Médicale Inserm	14	3.75	3,047	217.64	9	France
5	Universite Paris Saclay	12	3.22	3,005	250.42	8	France
6	Harvard Medical School	12	3.22	315	26.25	8	USA
7	Gustave Roussy	11	2.95	2,992	272	7	France
8	National Institutes of Health NIH USA	11	2.95	581	52.82	7	USA
9	Chinese Academy of Sciences	11	2.95	435	39.55	8	China
10	Shanghai Jiao Tong University	10	2.68	405	40.5	6	China

CPP, citations per publication.

**Figure 5:**
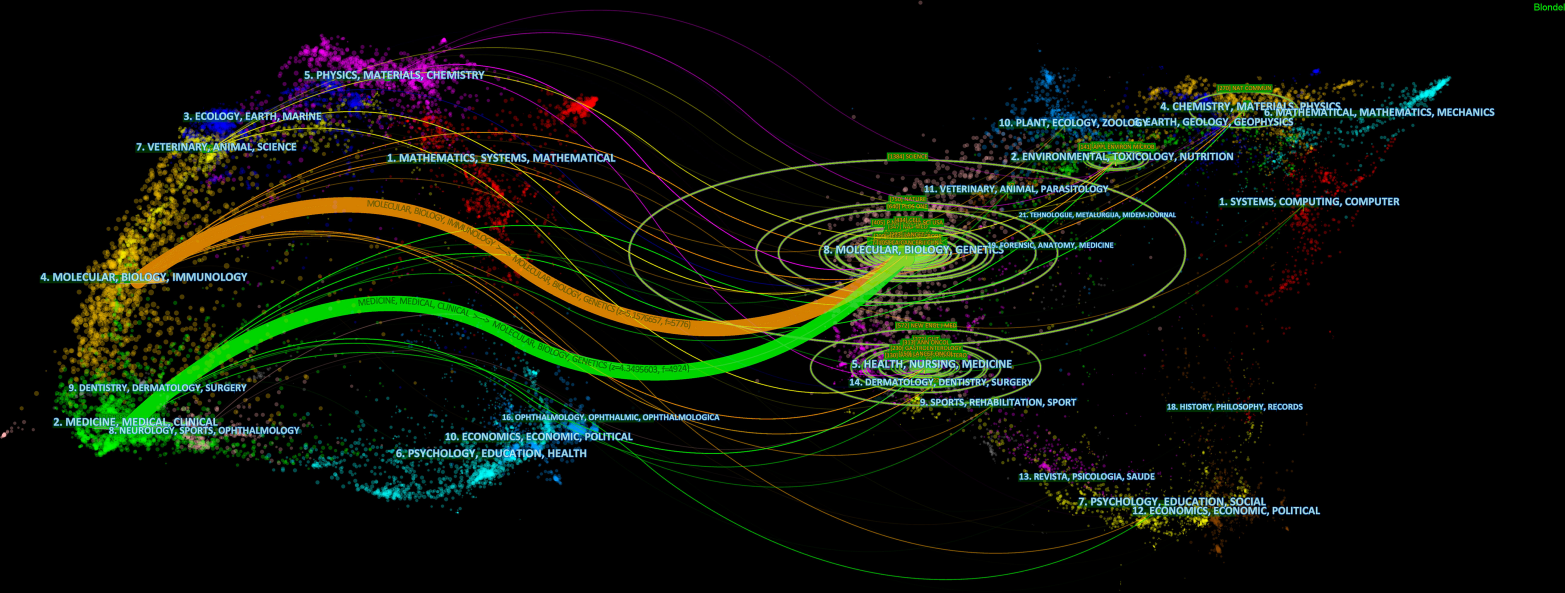
A dual-map overlay of journals related to the gut microbiota and lung cancer from 2011 to 2022.

The retrieved publications in this study were published in 225 journals, and the top 3 most productive journals were the *International Journal of Molecular Sciences* (4.02% of 373, with 15 papers), *Frontiers in Immunology* (3.75% of 373, with 14 papers), and *Cancers* (2.68% of 373, with 10 papers). Notably, as shown in **Table 2**, the journal of *Frontiers in Immunology* had both the highest IF (8.79) and the CPP (27.64), which indicated that it was considered the most pivotal journal in this field. The link between citing and cited journals is demonstrated in **Figure 5**, and the main citation paths were identified, including 1) molecular, biology, and immunology-molecular, biology, and genetics (z = 5.16, f = 5,776), and 2) medicine, medical, and clinical-molecular, biology, and genetics (z = 4.35, f = 4,924).

**Table 2:** The top 10 scholarly journals in the field of gut microbiota and lung cancer from 2011 to 2022.

Rank	Journal	Counts	% of 373	CPP	H-index	IF (2021)	Category quartile
1	*International Journal of Molecular Sciences*	15	4.02	5.27	4	6.21	Q1/Q2
2	*Frontiers in Immunology*	14	3.75	27.64	8	8.79	Q1
3	*Cancers*	10	2.68	6.5	5	6.57	Q1
4	*Frontiers in Oncology*	7	1.88	3.14	2	5.74	Q2
5	*BMC Cancer*	7	1.88	5	3	4.64	Q2
6	*Thoracic Cancer*	7	1.88	8.57	4	3.22	Q3
7	*Frontiers in Microbiology*	6	1.61	7.5	2	6.06	Q1
8	*Scientific Reports*	6	1.61	24.17	4	4.99	Q2
9	*Cancer Immunology Immunotherapy*	5	1.34	16.2	4	6.63	Q1/Q2
10	*Clinical & Translational Oncology*	5	1.34	5.6	3	3.34	Q3

CPP, citations per publication; IF, impact factor.

### Analysis of authors and co-authorship of authors

**Figure 6:**
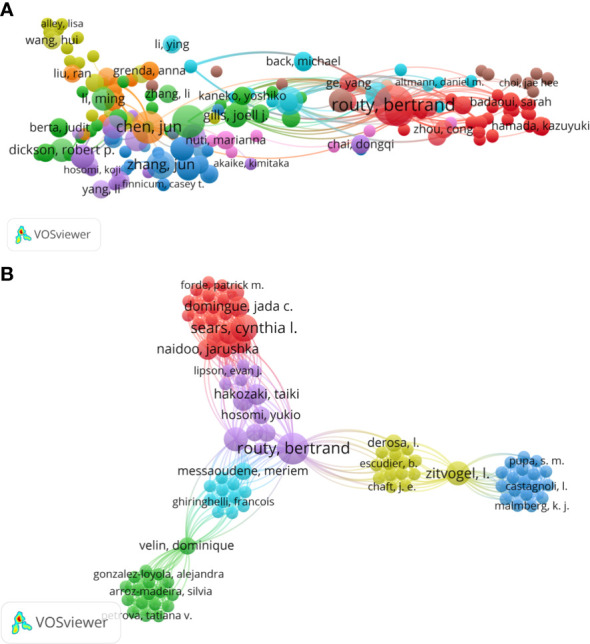
**(A)** The network map of co-citation between authors. **(B)** The co-authorship map of authors indicating the authors that cooperated in the field of gut microbiota and lung cancer from 2011 to 2022. Nodes represent authors, and larger nodes indicate a higher number of publications, the clusters were labeled using different colors, and the links represent author collaborations.

A total of 345 authors have contributed to this field. Routy Bertrand ranked first as the most prolific author with nine publications, followed closely by Zitvogel Laurence (seven publications), Derosa Lisa (six publications), and Richard Corentin (six publications). Meanwhile, Routy Bertrand had the highest H-index of 8 (**Table 3**). VOSviewer (Version 1.6.18) was used to analyze the co-cited authors identified from all publications in this study **(** **Figure 6A**). The top 10 co-cited authors are listed in **Table 3**. Routy Bertrand ranked first, with 668 citations, followed by Zitvogel Laurence (581 citations), Sears Cynthia L (141 citations), Richard Corentin (99 citations), and Jun Chen (85 citations). Additionally, the co-authorship map of authors, which indicated the authors that cooperate in this field over the past years, was generated, and the collaboration of 21 authors is shown in **Figure 6B**.

**Table 3:** The top 10 authors and co-cited authors in the field of gut microbiota and lung cancer from 2011 to 2022.

Rank	Author	Count	CPP	H-index	Co-cited author	Citations	Total link strength
1	Routy Bertrand	9	334.33	8	Routy Bertrand	668	56
2	Zitvogel Laurence	7	426.43	6	Richard Corentin	99	39
3	Derosa Lisa	6	486.67	5	Sears Cynthia L	141	38
4	Richard Corentin	6	406.33	5	Gills Joell J	30	35
5	Jun Chen	5	21.2	4	Shaikh Fyza Y	30	35
6	Pinato David J	5	77.6	5	Zitvogel Laurence	581	35
7	Okuma Yusuke	5	36.2	4	Back Michael	9	34
8	Sears Cynthia L	5	28.8	4	Boyle Frances	9	34
9	Botticelli Andrea	4	45	4	Carroll Susan	9	34
10	Arielle Elkrief	4	28.63	4	Jun Chen	85	34

CPP, citations per publication.

### Analysis of cited references

**Figure 7:**
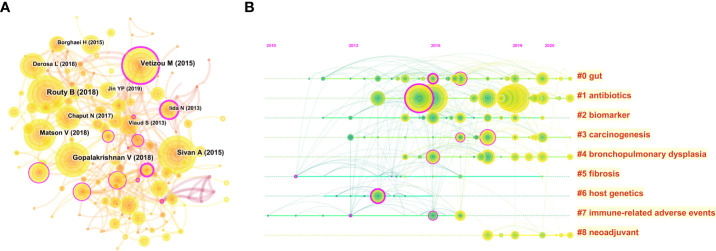
**(A)** CiteSpace visualization map of cited references in the field of gut microbiota and lung cancer from 2011 to 2022. The nodes represent cited references, and the lines between the nodes represent cited-reference relationships. **(B)** The timeline view clusters of co-cited references and their cluster labels *via* CiteSpace. The cluster with warmer colors and larger nodes contained more publications, indicating that this clustering issue was the hotspot in this field.

The visualization map of cited references consisted of 169 nodes and 381 links. Among them, the top 10 cited references in terms of citation frequency are shown in **Figure 7A**. Notably, the basic characteristics of these top 10 highest cited references are shown in **Table 4**. The top 3 publications with the highest citations were all published in *Science* (IF: 63.832), entitled “Gut microbiome influences efficacy of PD-1-based immunotherapy against epithelial tumors” by Routy Bertrand, which was the most cited reference with 2,339 citations (28), followed by “Commensal *Bifidobacterium* promotes antitumor immunity and facilitates anti-PD-L1 efficacy” with 2,069 citations (29) and “Gut microbiome modulates response to anti-PD-1 immunotherapy in melanoma patients” with 2,010 citations (30). Interestingly, the aforementioned publications are all dedicated to the correlation between the gut microbiome and the anti-tumor efficacy of PD-1, revealing that the gut microbiota could improve anti-tumor efficacy by modulating the tumor response to checkpoint blockade immunotherapy. Furthermore, the timeline view cluster of co-cited references related to the gut microbiota and lung cancer was generated by CiteSpace (**Figure 7B**), while the log-likelihood rate (LLR) was used to identify the distribution of hotspots from publications in the nine clusters. Specifically, the cluster with warmer colors and larger nodes contained more recent publications, indicating that the cluster was the hotspot in this field in recent years. Hence, as shown in **Figure 7B**, cluster #1 (antibiotics) has become a continuing hotspot of research in this field, with mapping of the impact of antibiotics on anti-tumor efficacy attracting great attention. In addition, cluster #0 (gut), cluster #3 (carcinogenesis), cluster #4 (bronchopulmonary dysplasia), and cluster #8 (neoadjuvant) indicated the hotspots in this field during the past latest years. To verify the credibility of the clusters, the structural characteristics of the cited reference clusters, especially silhouette, were established **(** **Table 5**). To the best of our knowledge, a silhouette value greater than 0.7 was generally considered very credible for the cluster (31). As shown in **Table 5**, the biggest cluster was “gut” with a size of 28, while cluster #6 (host genetics) ranked first in terms of silhouette. Additionally, cluster #1 (antibiotics) had the highest LLR, which contained 20 references with a rate of 11.02, followed by “biomarker” (8.16), “immune-related adverse events” (7.43), “fibrosis” (7.23), and “carcinogenesis” (6.37).

**Table 4:** The top 10 highly cited references in the field of gut microbiota and lung cancer from 2011 to 2022.

Rank	References	Journal	IF 2021	First author	Publication time	Total citations
1	Gut microbiome influences efficacy of PD-1-based immunotherapy against epithelial tumors	*Science*	63.832	Routy, B	2018	2,339
2	Commensal Bifidobacterium promotes antitumor immunity and facilitates anti-PD-L1 efficacy	*Science*	63.832	Sivan, A	2015	2,069
3	Gut microbiome modulates response to anti-PD-1 immunotherapy in melanoma patients	*Science*	63.832	Gopalakrishnan, V	2018	2,010
4	Anticancer immunotherapy by CTLA-4 blockade relies on the gut microbiota	*Science*	63.832	Vetizou, M	2015	1,838
5	The commensal microbiome is associated with anti-PD-1 efficacy in metastatic melanoma patients	*Science*	63.832	Matson, V	2018	1,368
6	Commensal Bacteria Control Cancer Response to Therapy by Modulating the Tumor Microenvironment	*Science*	63.832	Iida, N	2013	1,293
7	The Intestinal Microbiota Modulates the Anticancer Immune Effects of Cyclophosphamide	*Science*	63.832	Viaud, S	2013	1,226
8	Baseline gut microbiota predicts clinical response and colitis in metastatic melanoma patients treated with ipilimumab	*Annals of oncology*	51.769	Chaput, N	2017	602
9	Negative association of antibiotics on clinical activity of immune checkpoint inhibitors in patients with advanced renal cell and non-small-cell lung cancer	*Annals of oncology*	51.769	Derosa, L	2018	441
10	The Diversity of Gut Microbiome is Associated With Favorable Responses to Anti-Programmed Death 1 Immunotherapy in Chinese Patients With NSCLC	*Journal of thoracic oncology*	20.121	Jin, YP	2019	193

IF, impact factor.

**Table 5:** The structural characteristics of the cited-reference clusters.

Cluster ID	Size	Silhouette	Mean (year)	Cluster name (LLR)
#0	28	0.559	2015	Gut (6.13)
#1	20	0.753	2018	Antibiotics (11.02)
#2	16	0.705	2015	Biomarker (8.16)
#3	16	0.613	2016	Carcinogenesis (6.37)
#4	14	0.754	2016	Bronchopulmonary dysplasia (4.90)
#5	10	0.815	2013	Fibrosis (7.23)
#6	9	0.920	2013	host genetics (5.89)
#7	9	0.908	2013	Immune-related adverse events (7.43)
#8	7	0.848	2018	Neoadjuvant (4.34)

LLR, log-likelihood ratio.

### Analysis of keywords and co-occurrence clusters and burst

**Figure 8:**
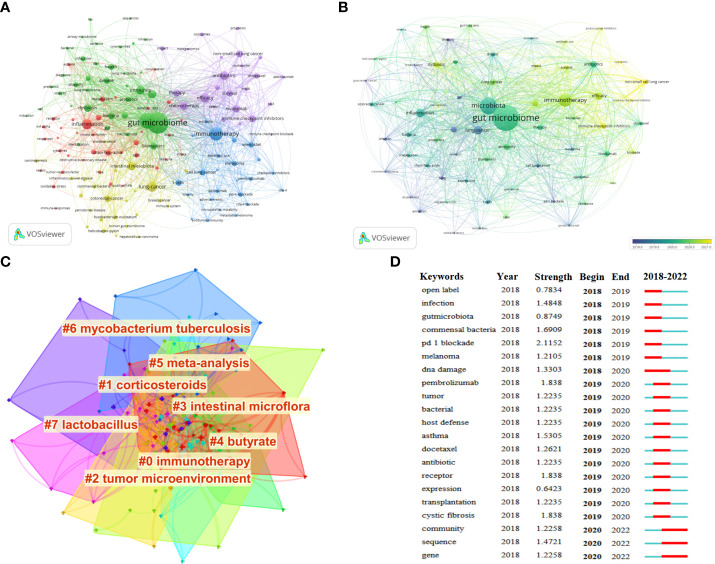
**(A)** Visualization of keyword co-occurrence analysis. The size of nodes indicates the frequency of occurrences of the keywords. The lines between the nodes represent their co-occurrence in the same publication. The shorter the distance between two nodes, the larger the number of co-occurrence of the two keywords. **(B)** Visualization map of the 64 keywords with a frequency of no less than 10 times since 2019 generated by using VOSviewer (blue, earlier; yellow, later). **(C)** The cluster of keywords related to the gut microbiota and lung cancer from 2011 to 2022. The different colors mean different clusters. **(D)** CiteSpace visualization map of the top 20 keywords with the strongest citation bursts from 2011 to 2022.

A total of 145 keywords were identified as occurring more than five times, which could be classified as five clusters (**Figure 8A**). Keywords, such as “gut microbiome” and “lung cancer”, were excluded. Meanwhile, “immunotherapy”, “inflammation”, “antibiotics”, “efficacy”, and “immune checkpoint inhibitors” were identified to a better perspective. Notably, the 64 keywords with a frequency of no less than 10 times since 2019 were identified for further analysis. As shown in **Figure 8B**, the more yellow-colored dots were the most recent keywords to appear, indicating that these were the latest research trends in this field, mainly including immunotherapy, immune checkpoint inhibitors, dysbiosis, survival, efficacy, and non-small cell lung cancer. Furthermore, a network map to visualize the clusters of keywords related to the gut microbiota and lung cancer from 2011 to 2022 was also constructed. According to **Figure 8C**, cluster #0 labeled “immunotherapy” was the largest cluster, followed by “corticosteroids” (cluster #1), “tumor microenvironment” (cluster #2), “intestinal microflora” (cluster #3), “butyrate” (cluster #4), “meta-analysis” (cluster #5), “mycobacterium tuberculosis” (cluster #6), and “lactobacillus” (cluster #7). The top 25 keywords with the strongest citation bursts in this field from 2018 were spotlighted, which was attributed to a significant increase in the number of publications since 2018. Among them, the top 5 burst strength keywords were defined, including “PD-1 blockade”, with the highest burst strength of 2.1152, followed by “pembrolizumab” (1.838), “receptor” (1.838), “cystic fibrosis” (1.838), and “commensal bacteria” (1.6909) (**Figure 8D**).

## Discussion

Currently, bibliometric analysis is increasingly used to detect the status and trends in a particular field. To date, bibliometric analyses of the gut microbiota and lung cancer have not been reported. Hence, this is the first study to prospect the trends of the impact of the gut microbiota on lung cancer, which may help provide an instructive perspective for future research.

## General information

The current analysis demonstrated a significant increase in the number of annual publications and citations in the field of gut microbiota and lung cancer, especially in 2018, reaching the highest in 2022, indicating that this field has received sustained attention from researchers in recent years. The highest H-index and the number of annual citations were in 2019 and 2021, respectively. Therefore, publications retrieved in this field from 2019 deserved further in-depth mining. Additionally, the H-index for the last 2 years was lower, contributing to the fact that 2021 and 2022 were very close to the time of data extraction for this study (25; October 2022).

A total of 60 countries/regions, 941 institutions, and 345 authors contributed to this field. Internationally, China ranked first in terms of the total number of publications outputs, which reflected the fact on the increasing number of scholars in China who are dedicated to this field. However, the CPP and H-index of China were lower than those of other countries, such as the United States, indicating that while the total of publications has increased in China, there was still a lack of high-quality articles. Notably, the United States remained the prominent academic driver in this field, with high academic status, as confirmed by the highest CPP and H-index. Moreover, according to the visual map of international collaboration, the United States sustained a close cooperative relationship with many countries engaged in this field, including China, Germany, and France.

Interestingly, half of the top 10 institutions were from France, indicating that France has emerged as a major center for research in this field. Except for China, the rest of the top 10 institutions were from developed countries, which manifested that significant lagging in developing countries existed in this field. Therefore, Chinese institutions should actively maintain and benefit from close cooperation with affiliated institutions from developed countries to increase their international influence in future research strategies. Among the analysis of authors and co-cited authors, Routy Bertrand from Canada was the most active scholar and the most co-cited author, indicating that his research has had an important impact on the development of this discipline. Specifically, Routy Bertrand mainly focused on the effects of the gut microbiota on immune checkpoint inhibitors and carried out several clinical studies on the gut microbiota in the treatment of lung cancer (28, 32), confirming that the gut microbiota has an important therapeutic role in lung cancer.

## Evolution of research hotspots and frontiers

The research hotspots and frontiers in the field of gut microbiota for lung cancer were clarified by analyzing a combination of the highest cited references, keyword co-occurrence, clusters, and burst, which mainly included the gut microbiota directly affected the efficacy of immunotherapy for lung cancer. Immunotherapy is a crucial therapy in the comprehensive treatment of lung cancer. Recently, several immune check inhibitors (ICIs), such as anti-PD-1/PD-L1/CTLA-4, have been widely applied in lung cancer, which can effectively improve progression-free survival in patients with lung cancer (29, 33, 34). Furthermore, the gut microbiota improves the effects of anti-tumor by modulating the tumor response to checkpoint blockade immunotherapy (35). Notably, increasing evidence seemed to suggest that the efficacy of immunotherapy is influenced by relevant immune checkpoints, such as PD-1/PD-L1/TMB, which may be closely associated with alterations in the gut microbiota, including *Bifidobacterium longum*, *Collinsella aerofaciens*, and *Enterococcus faecium* (28, 36). Therefore, regulating the gut microbiota may be an important method to upgrade the efficacy of immunotherapy in lung cancer patients. Moreover, multiple basic studies have reported that the gut microbiota affected the therapeutic effect of chemo-radiotherapy in lung cancer (37, 38), suggesting that modulating the gut microbiota was an important way to improve chemo-radiotherapy sensitivity. However, only two clinical trials of immunotherapy combined with probiotics for lung cancer were searched, with the registration number NCT04699721/NCT05094167. Thus, randomized, controlled clinical trials on the combination of regulatory microbiota (including probiotics and fecal microbiota transplantation) and anti-tumor therapies (such as chemotherapy, radiotherapy, and immunotherapy) with a fixed and standardized research protocol and strict quality control are urgently warranted.

Then, the application of different gut bacteria in lung cancer was another emerging topic identified by analyzing the keyword clusters, wherein the *Lactobacillus* obtained the most attention. Studies have demonstrated that *Lactobacillus*, one of the most widely studied probiotics in lung cancer, can significantly inhibit metastasis of tumor cells to the lung, thereby improving the prognosis of patients with lung cancer (39, 40). A previous study by Valentino Le Noci et al. suggested that an increase in the gene encoding the joining chain (J chain) of immunoglobulins was observed by *Lactobacillus* aerosolization, while high levels of J-chain mRNA strongly associated with a good prognosis for patients with lung adenocarcinoma (41). In addition, intravenous and intradermal injections of *Lactobacillus casei* significantly increased the anti-tumor activity in lung cancer model mice (42). However, uncertainty about the efficacy of *Lactobacillus* is one of the preeminent reasons limiting clinical application. Therefore, to address this issue, the combination of *Lactobacillus* with other probiotics, such as *Bifidobacterium*, may provide more stable and excellent efficacy, which is helpful for guiding new research directions of different genera in the treatment of lung cancer in the future.

The final topic was the hotspots and trends of the mechanism of the gut microbiota on lung cancer progression. The gut microbiota may be involved in the occurrence and development of lung cancer through the following potential ways. First, changes in diversity and abundance, which are labeled dysbiosis, may be associated with the occurrence of lung cancer (43). Studies revealed that there were significant differences in the gut microbiota beta diversity between patients with lung cancer and healthy controls, mainly manifested by the increased abundance of *Enterococcus* and the decrease in the levels of the phylum Actinobacteria and genus *Bifidobacterium* (44, 45). Notably, these gut microbiota may be potential biomarkers of lung cancer, which would provide clues in the assessment of lung cancer progression and the effective development of targeted therapy (46). Second, gut microbiota dysbiosis promoted the release of inflammatory mediators, along with the release of multiple toxins that promote the production of free radicals, which contributed to the occurrence of lung cancer (47). Recently, the regulation of metabolites mediated by the gut microbiota on lung cancer progression was a hotspot in this field. Several studies have found that butyrate-producing bacteria decreased significantly in lung cancer patients, while promoting the levels of butyrate mediated by the gut microbiota played anti-inflammatory, anti-tumor proliferation, and metastasis roles (48–50). Hence, the gut microorganism-mediated metabolic network pathway is the key mechanism to revealing lung cancer and the gut microbiota.

## Limitations

Despite the strict principles of bibliometric analysis being adhered to in this study, there are some unavoidable limitations. First, only articles and reviews published within a specific period of time in the WoSCC were included, thereby potentially leading to publication bias in the study results. However, the search timespan of this study was sufficiently long for the findings, which can reflect the research trends in this field of interest. Second, a series of strict principles were identified in the early stages of publication retrieval, while two individuals were selected to review the publications initially searched so that many non-compliant documents were eliminated. However, bias in the selection of publications was difficult to avoid. Notwithstanding, we believe that our findings provide a relatively comprehensive overview of the gut microbiota and lung cancer.

## Conclusion

This bibliometric study provides an overview of the major research hotspots and frontiers in the field of gut microbiota and lung cancer, which provided potential collaborators and institutions, and hot topics. Specifically, the number of publications in this field has grown rapidly over the past decade. China has the largest number of publications in this field, while the United States and France have a greater influence. It is necessary to further strengthen cooperation among countries, especially emerging countries. The clinical and mechanism research of the gut microbiota in immunotherapy of lung cancer is currently a hotspot in this field. Although many studies have confirmed that the modulation of the gut microbiota can treat lung cancer and improve anti-tumor efficacy, there are few clinical trials in this field, especially in lung cancer patients with radio-chemotherapy. Therefore, more in-depth studies on the clinical efficacy and safety examination of the gut microbiota in lung cancer patients are needed. The findings of this study provide new perspectives on the field, which may shed light on a beneficial impact on further etiological studies, diagnosis, and treatment of lung cancer.

## Data availability statement

The raw data supporting the conclusions of this article will be made available by the authors, without undue reservation.

## Author contributions

HC, YL, and QY designed the study. HC, YL, and CY were responsible for data collection. HC, YL, CY, CW, and JZ were responsible for the investigation and construction of figures and tables. HC, and YL drafted the manuscript. QY revised and approved the final version of the manuscript.
